# Endocytic Rabs Are Recruited to the *Trypanosoma cruzi* Parasitophorous Vacuole and Contribute to the Process of Infection in Non-professional Phagocytic Cells

**DOI:** 10.3389/fcimb.2020.536985

**Published:** 2020-10-29

**Authors:** Betiana Nebaí Salassa, Juan Agustín Cueto, Julián Gambarte Tudela, Patricia Silvia Romano

**Affiliations:** ^1^Laboratorio de Biología de Trypanosoma cruzi la célula hospedadora, Instituto de Histología y Embriologìa, Consejo Nacional de Investigaciones Científicas y Técnicas (IHEM-CONICET), Universidad Nacional de Cuyo, Mendoza, Argentina; ^2^Facultad de Odontología, Universidad Nacional de Cuyo, Mendoza, Argentina; ^3^Instituto de Fisiología, Facultad de Ciencias Médicas, Universidad Nacional de Cuyo, Mendoza, Argentina; ^4^Instituto de Bioquímica y Biotecnología, Facultad de Ciencias Médicas, Universidad Nacional de Cuyo, Mendoza, Argentina; ^5^Facultad de Ciencias Médicas, Universidad Nacional de Cuyo, Mendoza, Argentina

**Keywords:** *Trypanosoma cruzi*, host cell infection, endocytosis, Rab proteins, *T. cruzi* parasitophorous vacuole

## Abstract

*Trypanosoma cruzi* is the parasite causative of Chagas disease, a highly disseminated illness endemic in Latin-American countries. *T. cruzi* has a complex life cycle that involves mammalian hosts and insect vectors both of which exhibits different parasitic forms. Trypomastigotes are the infective forms capable to invade several types of host cells from mammals. *T. cruzi* infection process comprises two sequential steps, the formation and the maturation of the *Trypanosoma cruzi* parasitophorous vacuole. Host Rab GTPases are proteins that control the intracellular vesicular traffic by regulating budding, transport, docking, and tethering of vesicles. From over 70 Rab GTPases identified in mammalian cells only two, Rab5 and Rab7 have been found in the *T. cruzi* vacuole to date. In this work, we have characterized the role of the endocytic, recycling, and secretory routes in the *T. cruzi* infection process in CHO cells, by studying the most representative Rabs of these pathways. We found that endocytic Rabs are selectively recruited to the vacuole of *T. cruzi*, among them Rab22a, Rab5, and Rab21 right away after the infection followed by Rab7 and Rab39a at later times. However, neither recycling nor secretory Rabs were present in the vacuole membrane at the times studied. Interestingly loss of function of endocytic Rabs by the use of their dominant-negative mutant forms significantly decreases *T. cruzi* infection. These data highlight the contribution of these proteins and the endosomal route in the process of *T. cruzi* infection.

## Introduction

The Ras-associated binding proteins, Rabs, are small GTP–binding proteins that control the budding, transport, docking, and tethering of vesicles from a donor to an acceptor compartment. Early endocytosis is regulated by Rab5 subfamily of GTPases mainly composed by Rab5, Rab22a, and Rab21 (Li, 2012). Rab5, the first characterized protein and the prototype of this subfamily, is located in the cytoplasmic side of plasma membrane, endocytic vesicles and early endosomes (EE) and regulates the formation, uncoating, and transport of endocytic vesicles and fusion with early endosomes (Chavrier et al., 1990; Gorvel et al., 1991). Rab22a and Rab21 have similar distribution than Rab5 and promote, with it, the sorting to late endosomes (LE)/lysosomes due to interaction with similar effectors and regulators. Interestingly, the effector of Rab22a, Rabex-5, is a GEF of Rab5 and Rab21, indicating that these Rabs can also cooperate to produce the Rab22a-Rab5/Rab21 cascade (Zhu et al., 2009), which allow the sorting to LE. Moreover, the class C VPS/HOPS complex (Rieder and Emr, 1997; Seals et al., 2000), is a Rab5 effector that promotes the arrival of Rab7 and the sorting of endocytosed ligands from early to late endosomes which, in turn, will mature into lysosomes, the degradative compartment of the cell (Rink et al., 2005). Besides Rab7, other Rabs participate in the late endocytic transport. Rab39a is a poorly characterized Rab located in endocytic compartments that regulates transport and fusion of vesicles from the Golgi complex to LE/multivesicular bodies (Chen et al., 2003; Gambarte Tudela et al., 2019). In contrast, the so-called anterograde transport to plasma membrane is regulated by another subset of Rabs. Rab1 and Rab2 participate in the transport of secretory proteins throughout the endoplasmic reticulum and Golgi complex, while Rab3 is associated with the fusion of secretory vesicles to the plasma membrane, especially synaptic vesicles (Schlüter et al., 2004). In addition, the recycling of cargo from endosomes to plasma membrane is regulated by Rab4 and Rab11, which promote the rapid or the slow recycling of endocytic components, respectively (Grant and Donaldson, 2009). Due to antibodies against Rab proteins have low sensitivity, the most important tool used to study these proteins has been the expression of mutant proteins defective in the binding (dominant-negative mutants) or in the hydrolysis (dominant-positive mutants) of GTP. These mutants fused to GFP have been widely used to localize and to describe the function of a particular Rab in a particular traffic (Tisdale et al., 1992; Zerial and Stenmark, 1993).

*Trypanosoma cruzi*, the causal agent of Chagas disease, is an obligate intracellular parasite in mammalian hosts. The infective forms of *T. cruzi* are metacyclic or blood trypomastigotes, according to their origin from the feces of the insect vector or in the blood of mammalian hosts, respectively. After the invasion, trypomastigotes temporally reside in a membrane-bound compartment called the *T. cruzi* parasitophorous vacuole (TcPV). Inside the TcPV, trypomastigotes start the differentiation to amastigotes and the process ends in the cytoplasm. Amastigotes then actively replicate and when they are in high number, differentiate back to trypomastigotes which exit the cell to infect new cells and to continue the parasite cycle. Target cells are preferentially muscle cells like smooth muscle digestive tract and cardiac tissue, both places where *T. cruzi* develops the pathology.

Invasion of *T. cruzi* and transit in the TcPV is a key step during the *T. cruzi* intracellular cycle. Like other successful intracellular microorganisms, *T. cruzi* manipulates the host cell machinery to enter and persist within the host cell. Two main processes of invasion of trypomastigotes in non-professional phagocytes have been described up to date. In the well-described lysosomal-dependent model, *T. cruzi* exploits the plasma membrane damage repair response to invade the host cells. Trypomastigotes induce small injuries in the host cell membrane leading to a cytosolic calcium increase that in turn, elicits the exocytosis of lysosomes and entry of parasites (Tardieux et al., 1992; Rodríguez et al., 1996; Andrade and Andrews, 2005). However, in the lysosomal-independent model, trypomastigotes enter to the host cells by invagination of the plasma membrane and formation of a vacuole initially enriched in plasma membrane markers that later interact with lysosomes (Woolsey et al., 2003). Further studies from the same authors showed that a fraction of the nascent vacuole acquired EEA-1, a Rab-5 effector involved in the fusion of vesicles to early endosomes (Christoforidis et al., 1999). They postulated that this resultant vacuole gradually maturates until fusion with lysosomes (Woolsey and Burleigh, 2004).

It is widely accepted that fusion of TcPV with lysosomes is a key step to retain the parasite inside the cell (Andrade and Andrews, 2004). In a previous work from our lab we demonstrated the involvement of the SNARE VAMP7 in this process (Cueto et al., 2017). Eventhough the molecular components that regulate the transit of the initial vacuole until the fusion with lysosomes were not completely known up to date. Indeed, despite evidences that showed the presence of Rab5 and Rab7 in the TcPV (Wilkowsky et al., 2002; Maganto-Garcia et al., 2008; Barrias et al., 2010), the functional role of them in the *T. cruzi* infection still remains not understood. In this work we explored the possible participation of the main vesicular pathways that would contribute to the control of the early events during *T. cruzi* infection, mainly focusing in the mechanisms that promote vacuole maturation and lysosomal fusion.

## Methods

### Reagents

Minimal essential medium (α-MEM) and Dulbecco–modified minimal essential medium (D–MEM) were obtained from Gibco Laboratories (Buenos Aires, Argentina). Fetal bovine serum (FBS) was purchased from Natocor S.A. (Córdoba, Argentina). Rabbit anti–*T. cruzi* polyclonal antibody was kindly provided by Dr. Catalina Alba (Instituto de Investigaciones en Microbiología y Parasitología Médica, Buenos Aires, Argentina). The secondary Cy3 conjugated anti–rabbit antibody was purchased from Life Technologies (Buenos Aires, Argentina).

### Plasmids

pEGFPC3 encoding GFP–VAMP3 and GFP–VAMP7 were previously characterized by Dr. Thierry Galli (The École des Neurosciences de Paris Île-de-France, Paris, France) (Martinez-Arca et al., 2000a,b) and are available at Addgene. pEGFP-Rab1 WT, pEGFP-Rab3a WT, pEGFP-Rab4 WT, pEGFP-Rab6 WT, pEGFP-Rab25 WT, pEGFP-Rab22a WT, and pEGFP-Rab22a S22N were kindly provided by Dr. Javier Magadán (Instituto de Histología y Embriología de Mendoza “Dr. Mario Burgos,” Mendoza, Argentina) (Magadán et al., 2006). Plasmids encoding enhanced GFP (EGFP)-Rab7 and its mutants were kindly provided by Bo van Deurs (University of Copenhagen, Copenhagen, Denmark) and the plasmid encoding enhanced GFP (EGFP)-Rab5wt was kindly provided by Dr. Philip D. Stahl (Washington University). The pEGFP-Rab29 was gently provided by Drs. Jorge Galán and Stefania Spano (Yale University School of Medicine, New Haven). The pEGFPRab11 plasmid was used as previously described (Savina et al., 2002). pcDNA.DEST47-GFP-Rab39a WT and pcDNA.DEST47-GFP-Rab39a S22N were a generous gift from Bruno Goud (Curie Institute, Paris, France). pEGFP-Rab21 WT and pEGFP-Rab21 T31N were gifted by Dr. Thierry Galli (The École des Neurosciences de Paris Île-de-France, Paris, France).

### Cell Culture

Vero cells, a monkey epithelial cell line (obtained from ABAC, Asociación Banco Argentino de Células, Buenos Aires, Argentina) were grown in D–MEM supplemented with 10% FBS and antibiotics at 37°C in an atmosphere of 95% air and 5% CO_2_. CHO cells (ABAC), were maintained in α-MEM supplemented with 10% FBS and antibiotics at 37°C in an atmosphere of 95% air and 5% CO_2_.

### Propagation of *T. cruzi* Trypomastigotes

Y strain of *T. cruzi* was provided by Dr. Wanderley De Souza (Instituto de Biofísica Carlos Chagas Filho, Universidade Federal do Rio de Janeiro, Brasil) and handled in a biosafety level II facility. Tissue cell trypomastigotes (TCT) was prepared as follows. Vero cells (5 × 10^5^ cells/ml) were plated in T25 flasks and maintained at 37°C in D–MEM supplemented with 3% FBS and antibiotics (infection medium). Cells were infected with TCT suspensions (5 × 10^6^ cells/ml) for 3 days in infection medium at 37 °C in an atmosphere of 95% air and 5% CO_2_. After 4 to 6 days, intracellular TCT lysed the cells and reached the medium. Medium containing parasites was harvested and centrifuged at 600 g for 15 min at room temperature. The supernatant was discarded, and the pellet, containing TCT and amastigotes, was covered with 1 ml of fresh medium and incubated for 3 h at 37°C to allow TCT to swim up. Supernatant enriched in TCTs was harvested, and parasites were counted in a Neubauer chamber and used for infection experiments. All procedures involved live *T. cruzi* were made under a biosafety level II and approved by the institutional biosecurity committee.

### Cell Transfections

The previous day, CHO cells were plated on 13 mm round coverslips distributed in 24 well-plates in α-MEM supplemented with 10% FBS and antibiotics. Then cells 90% of confluence (about 1,50,000 cells), were transfected with plasmids (1 μg/μl) using the Lipofectamine 2000 reagent (Thermo Fisher Scientific) according to the instructions of the manufacturer. Transfected cells were incubated for 24 h in an appropriate medium before being exposed to parasites. After that, cells were infected as follow.

### Infection of Cells With Trypomastigotes

CHO cells attached to coverslips in 24 well-plates were washed three times with PBS. Then a TCT–enriched suspension was added at a multiplicity of infection of 20. To favor parasite–cell interaction, plates were subsequently centrifuged for 5 min at 4°C. Infection was carried out for 1 h at 37°C (or 15 min in a one-time point in kinetic experiments). Afterward, extracellular parasites were removed by washing several times, and cells were subjected to IF.

### Immunofluorescence

Cells were fixed with 4% paraformaldehyde solution in PBS for 20 min at room temperature and subsequently quenched by incubating with 50 mM NH_4_Cl in PBS. Then, cells were permeabilized with 0.1% saponin in PBS containing 0.2% BSA and incubated with primary antibodies for 2 h. Intracellular parasites were detected with a rabbit anti–*T. cruzi* polyclonal antibody (1:200). Next, cells were incubated for 2 h with Cy3 anti–rabbit (1:500). Additionally, cells were treated with Hoechst for DNA staining, mounted onto glass slides with Mowiol and analyzed with an Olympus Confocal Microscope FV1000–EVA (Olympus), with the FV10–ASW (version 01.07.00.16) software. In some experiments we control the immunofluorescence by performing a double detection prior and after permeabilization using the same primary antibody and two secondary antibodies labeled with different fluorescent markers. After the analysis of images, we confirmed that only the intracellular parasites were stained after permeabilization whereas extracellular parasites exhibit double-labeling.

### Analysis of Protein Recruitment to TcPV

The recruitment of Rabs to TcPV was confirmed by studying the fluoresce profile in the site where the *T. cruzi* vacuole is located. In the confocal image depicting the infected cell, a straight line was drawn through the vacuole and fluorescence intensity across this line were visualized in a graphic. If the protein was recruited, it can be seen how the fluorescence intensity of the green channel (Rab protein) on the surface of TcPV is several times greater than the fluorescence intensity of cytoplasm (background). Recruitment was considered positive when the fluorescence intensity of the area of interest was at least three times greater than the surrounding fluorescence. The TcPV can be localized by the increase in the intensity of red fluorescence that correspond to parasite detection and also by the blue fluorescence that labeled the parasite nucleus. When the protein under study was not recruited to the vacuole membrane, the green peaks around the parasite were not observed in the graphic. After analysis of recruitment, the percentage of vacuoles with positive recruitment were calculated for each Rab protein and compared with the vector.

## Results

### Rab Proteins From Early and Late Endocytic Pathway Are Recruited to the TcPV

**Figure 1:**
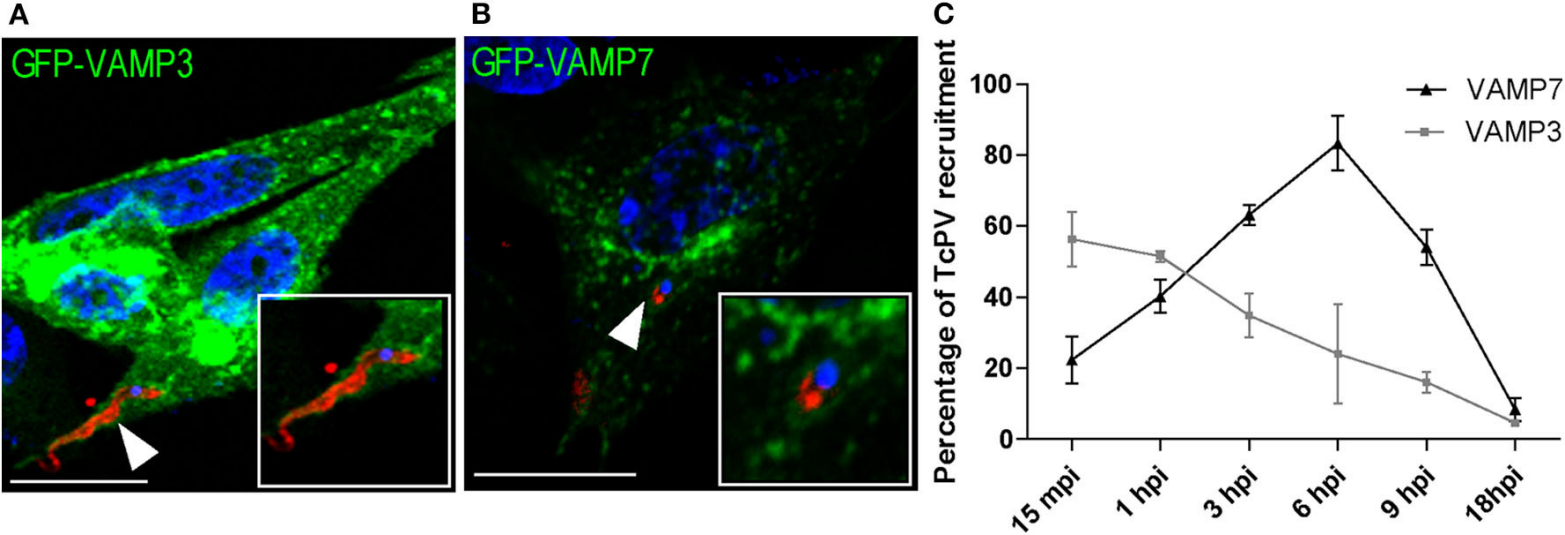
Kinetics of the recruitment of VAMP3 and VAMP7 on the *T. cruzi* parasitophorous vacuole. **(A,B)** CHO cells overexpressing GFP-VAMP3 or GFP-VAMP7 were infected with TCT (MOI 20) for 15 min or 6 h, respectively. The parasites were detected by indirect immunofluorescence using a specific antibody anti-*T. cruzi* followed by a secondary antibody labeled with Cy3 (red). The DNA of nuclei and kinetoplasts were detected with Hoechst (blue). Confocal images are representative for each condition and the magnifications are delimited in the original photo. Scale bar: 10 μm. **(C)** The percentage of the recruitment of VAMPs to TcPV were quantified from the images obtained by confocal microscopy at the indicated times after infection from 15 min to 18 h. Data are representative of three independent experiments.

In a previous work we studied the recruitment of the SNARE proteins VAMP3 and VAMP7 to the parasitophorous vacuole of *T. cruzi* CL Brener strain at different times after infection. In that work we established that formation of the vacuole is extended up to 1 h after infection followed by vacuole maturation which was characterized by the arrival and fusion of VAMP7 and Lamp1-positive vesicles and vacuole acidification from 1 h to around 6 h after infection (Cueto et al., 2017). In this work we used the *T. cruzi* Y strain, we studied the recruitment of these SNAREs and confirmed their presence in the vacuole membrane (Figures 1A,B). We also observed a similar kinetics of association displayed by these proteins although the transit in the vacuole was slower than the previously observed for CL Brener strain showing the peak of VAMP7 recruitment at 6 h after infection (Figure 1C). To characterize the possible participation of endocytic, secretory and recycling pathways in the *T. cruzi* entry, we studied the presence of different Rabs, representative of these routes, at 1 h after infection (1 hpi) which is, according to our data, the time between formation and maturation of the vacuole. To asses this, different Rab proteins fused with GFP were overexpressed in CHO cells before infection with TCT. At 1 hpi, cells were fixed and parasites were detected by indirect immunofluorescence followed by confocal microscopy analysis. We studied the recruitment of Rab5, Rab22a, and Rab21 representative of early endocytic transport and Rab7 and Rab39a from the late endosomes and lysosomes. Recycling pathways were analyzed by the presence of Rab4, Rab11, and Rab25 and exocytic/secretory route by Rab1, Rab3, Rab6, and Rab29 (Wandinger-Ness and Zerial, 2014). From the 12 Rabs analyzed, only five, Rab22a, Rab5, Rab21, Rab7, and Rab39a were significantly recruited to the vacuole membrane at this time (Table 1). These data showed that early and late endocytic compartments interact with the TcPV during its transit. In contrast, neither recycling nor secretory vesicles reach the vacuole at this time (Table 1; Supplementary Figure 1A).

**Table 1:** Analysis of the recruitment of Rab proteins to TcPV.

**TcPV recruitment**	**1 hpi**
Rab1	–
Rab3a	–
Rab4	–
Rab5	+ (***)
Rab6	–
Rab7	+ (***)
Rab11	–
Rab21	+ (***)
Rab22a	+ (***)
Rab25	–
Rab29	–
Rab39a	+ (**)

CHO cells overexpressing GFP-Rabs were infected by 1 h (1hpi), fixed and subjected to indirect immunofluorescence to detect parasites before confocal microscopy analysis. The number of parasites that showed recruitment of GFP-proteins to the parasitophorous vacuole was detected. The + and – signs represents the Rab proteins that were significantly or not significantly recruited to TcPV compared to GFP-Vector respectively. Data are representative of three independent experiments. (***P < 0.001, **P < 0.01, two-way ANOVA and Bonferroni's multiple comparison test).

### The TcPV Transit Is Characterized by the Sequential Acquisition of Endosomal Rabs

**Figure 2:**
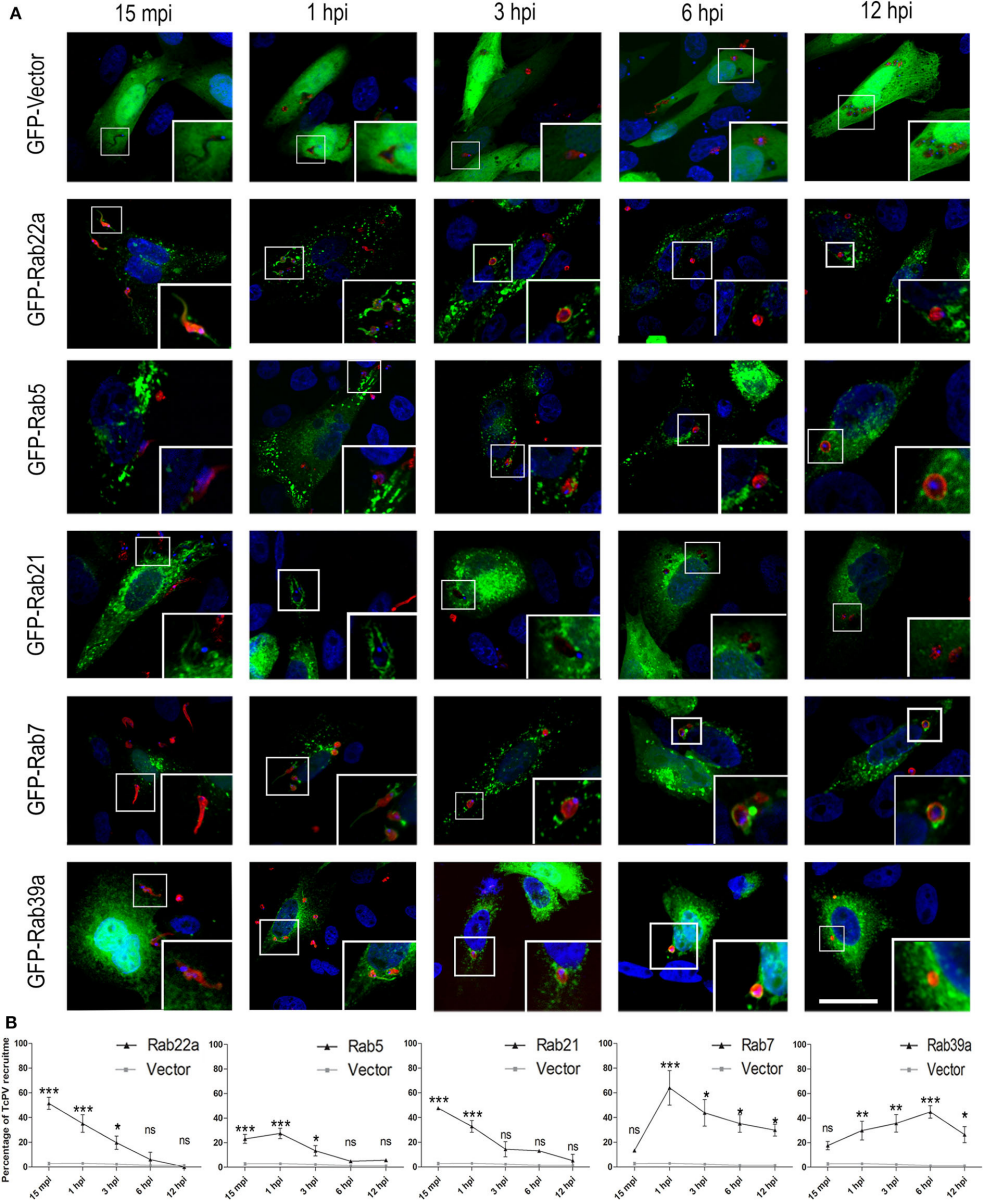
Recruitment kinetics of Rab proteins to *T. cruzi* parasitophorous vacuole. **(A)** Recruitment of Rab to TcPV were examined at the indicated time–points. CHO cells overexpressing GFP-Rabs were infected for 15 min (15 mpi), 1, 3, 6, or 12 h (hpi) with TCT (MOI 20). The parasites were detected by indirect immunofluorescence using a specific antibody anti-*T. cruzi* followed by a secondary antibody labeled with Cy3 (red). The DNA of nuclei and kinetoplasts were detected with Hoechst (blue). Images are representative for each condition and the magnifications are delimited in the original photo. Scale bar: 10 μm. **(B)** Kinetic graphs represent the recruitment of GFP-Rabs to TcPV. Data are representative of two independent experiments. (***P < 0.001, **P < 0.01, *P < 0.05, two-way ANOVA and Bonferroni's multiple comparison test).

To extend our characterization we next studied the kinetic of recruitment of the Rabs found in the TcPV. We performed infection experiments on cells expressing GFP-Rabs at different times from 15 min to 12 h and quantified the percentage of TcPVs surrounded by these proteins. We also used cells transfected with the GFP protein alone (GFP-Vector) as a control. Our data confirmed the presence of all Rabs found previously in the vacuole with some interesting qualitative and quantitative differences between them. Association of GFP-Rab5 to the vacuole membrane was discontinuous whereas the other Rabs displayed a uniform pattern around the parasite at the times of significant recruitment (Figure 2A). For early endocytic Rabs, the recruitment to TcPV was statistically significant at the early times mainly 15 min and 1 h and reached values around 50% of vacuoles for GFP-Rab22 and GFP-Rab21 in comparison with cells that overexpressed the vector (GFP alone) that never overcame ≈5% of vacuoles at any time studied. In contrast, Rab7 and Rab39a were significantly acquired at later times from 1 h up to even 12 h displaying a peak at 1 and 6 h, respectively (Figure 2B). Since the acquisition of VAMP7 indicates the arrival of lysosomes to the TcPV (Cueto et al., 2017), and considering that our data showed the presence of Rab7 prior to VAMP7 (compare Figure 1B with Figure 2B), this could indicate a key role of Rab7 in the docking and fusion of lysosomes, as was demonstrated previously (Bucci et al., 2000). We also analyzed the kinetic of recruitment of GFP-Rab11 and GFP-Rab6, representative of recycling and secretory routes, respectively, and obtained very low percentage of recruitments, similar to controls at all times (Supplementary Figures 1B,C). Therefore, these pathways displayed low (or null) interaction with the *T. cruzi*-containing vacuoles in the early stages of the infection.

### Endocytic Rabs Are Required for *T. cruzi* Infection

**Figure 3:**
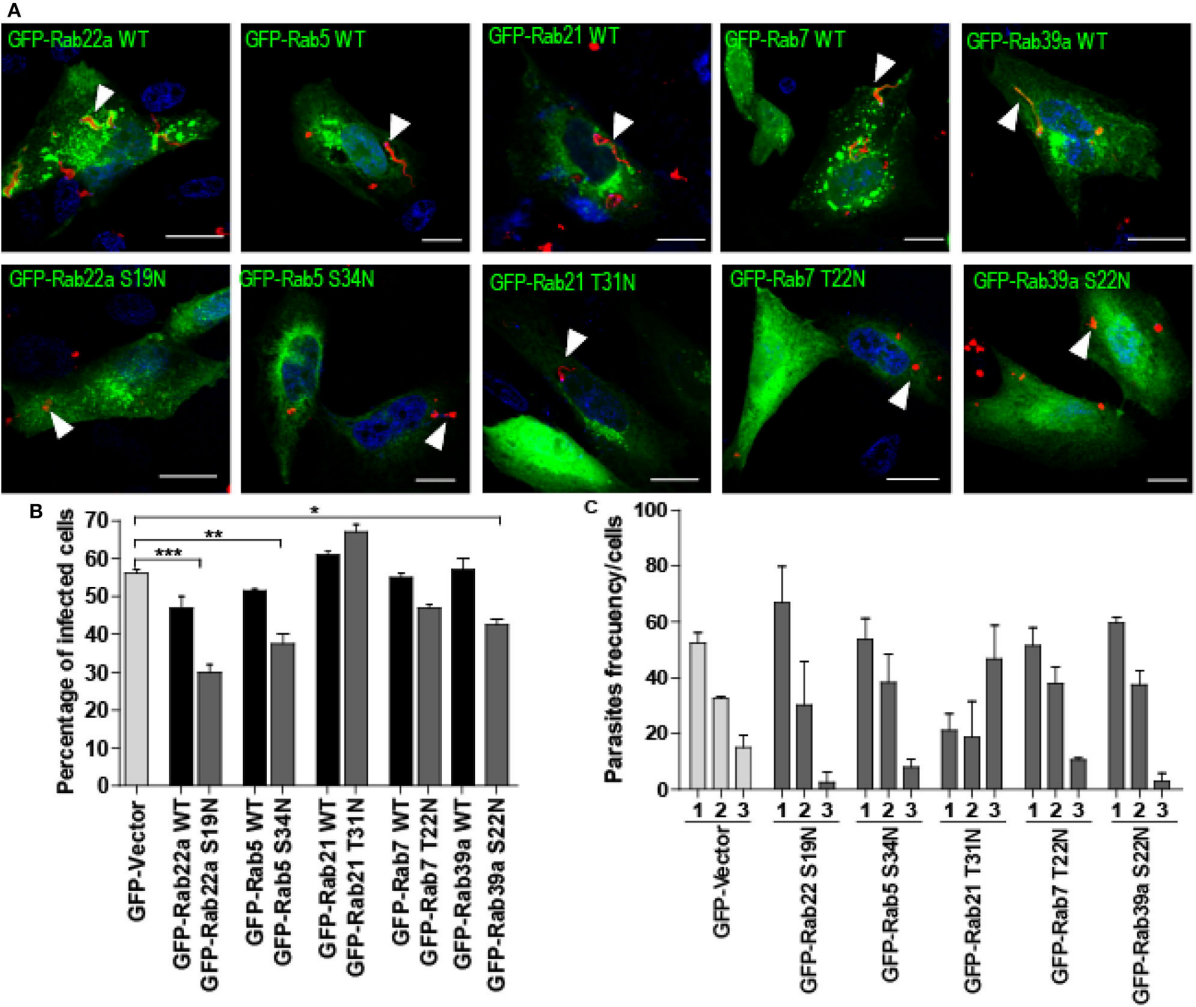
Effect of dominant-negative Rab-proteins on *T. cruzi* infection. **(A)** CHO cells overexpressing GFP-Rabs wild type (WT) or dominant-negative mutants were infected with TCT (MOI 20) for 1 h. After fixation, the parasites were detected by indirect immunofluorescence using a specific antibody anti-*T. cruzi* followed by a secondary antibody labeled with Cy3 (red). The DNA of nuclei and kinetoplasts were detected with Hoechst. Arrowheads indicate the localization of parasites. Scale bar: 10 μm. **(B)** Bar graph represents the percentage of infected cells at 1-h post-infection. For this experiment at least 120 cells were quantified. Data are representative of two independent experiments. (***P < 0.001, **P < 0.01, *P < 0.05, one-way ANOVA and Bonferroni's multiple comparison test). **(C)** Bar Graph represents the percentage of cells that contained 1, 2, or 3 or more parasites/cell at 1-h post-infection. For this experiment at least 120 cells were quantified. Data are representative of two independent experiments. (**P < 0.01, one-way ANOVA and Bonferroni's multiple comparison test).

To disclose the possible effect of endocytic Rabs on the process of *T. cruzi* infection, we transfected cells with the dominant-negative forms of these Rabs to prevent the normal function of the proteins and compare the level of infection obtained at 1 h after infection, with cells transfected with the GFP plasmid alone (GFP-Vector) and also with cells that overexpress the wild-type forms of these Rabs (GFP-Rab WT). Before infection assays, we tested the viability of dominant-negative transfected cells by the trypan blue dye exclusion staining and confirmed that these cells displayed the same viability than the controls (data not shown). Remarkably, in the infection experiments, we observed that in contrast to Rabs WT, the dominant-negative mutants of these proteins were not recruited to the TcPV membrane, therefore confirming their lack of activity (Figure 3A, arrowheads). Furthermore, with the exception of Rab21 mutant, the reduction of Rab function decreased the percentage of infected cells being this value significantly minor for Rab22a S19N, Rab5 S34N, and Rab39a S22N while the WT forms exhibit similar levels of infection in comparison with the GFP-Vector transfected cells (Figure 3B). We also quantified the percentage of mutant cells that contained 1, 2, or 3 (or more) parasites/cell and observed that the percentage of cells with 3 or more parasites was reduced to half (or even less) with the concomitantly increase in the percentage of cells with 1 or 2 parasites (Figure 3C). Unexpectedly, Rab21 T31N displayed an opposite result which could be explained by the different features of this mutant previously reported (Simpson et al., 2004; Egami and Araki, 2009). In summary these data confirmed that *T. cruzi* infection is partially regulated by the recruitment of endocytic Rabs to the TcPV which follows a maturation process until the arrival of lysosomes.

## Discussion

In previous works Woolsey and coworkers proposed that *T. cruzi* invasion initiated by a common step involving signaling, actin disassembly and plasma membrane invagination. This process culminated with the formation of a plasma membrane-derived vacuole that can directly fuse with lysosomes or pass through an early endosomal intermediate before lysosomal fusion (Woolsey et al., 2003; Woolsey and Burleigh, 2004). In agreement to these observations, our data confirmed the existence of this endosomal intermediate that follow a maturation process characterized by the sequential acquisition of endocytic Rabs. Rab22a, Rab5, and Rab21 were mainly recruited to vacuole at early stages (15 min-1 h) followed by Rab39a and Rab7 at later times. According to this model, the initial step of *T. cruzi* invasion takes very few minutes and involves the events that culminate with the formation of the nascent vacuole containing plasma-membrane components such as PI(4,5)P2. Interestingly, the work of Fernandes et al. demonstrated that *T. cruzi* induces the arrival of lysosomes containing sphingomyelinase that repairs the plasma membrane and generates a ceramide-enriched endosome that contains the recently internalized parasites (Fernandes et al., 2011).

In the second step, the initial vacuole will mature by the direct fusion with lysosomes or with endosomes which in turn will reach to lysosomes too. Why *T. cruzi* employs two different routes that finally converge in lysosomes is an open question to date. Due to in the steady state, many cells usually contain a relatively low number of lysosomes (Xu and Ren, 2015), and considering that lack of lysosomal fusion to vacuole leads to the previously described reversal infection (Andrade and Andrews, 2004), it is likely that the alternative endosomal route was an adaptation of parasites which could modify the membrane of their vacuole with endosomal proteins to prevent a new fusion with the plasma membrane and the exit of trypomastigotes from the cell. In agreement to this, our previous work demonstrated a significant increase on the infection produced by the expansion of the lysosomal compartment induced in pre-starved cells (Romano et al., 2009). The interaction with endosomes previous to lysosomes could also benefit some *T. cruzi* strains by conferring an additional time to be ready for the lytic lysosomal environment. Interestingly, a previous work showed that interaction of *T. cruzi* with cardiomyocytes leads to a reduction of Rab7 expression and impairment of the fusion between endocytic compartments (Batista et al., 2006). This response could be induced by soluble factors secreted from the parasite in an attempt to delay the fusion with lysosomes, a strategy displayed by several intracellular pathogens (Kumar and Valdivia, 2009).

Chronological acquisition of Rabs in the TcPV observed in our kinetic studies, could be related to the existence of a Rab cascade, described in other types of transports (Rink et al., 2005; Rivera-Molina and Novick, 2009). According to our kinetics experiments, it is possible that Rab22a, which is highly recruited at 15 min, allow the recruitment of Rab5 and/or Rab21 that in turn, by the action of specific effectors, recruits Rab7 that will promote the fusion with lysosomes. Fusion with early endosomes confer to TcPV some differential properties compared to the extracellular site where the parasites come from. Acidification of TcPV is crucial to the action of the Tc-Tox, the *T. cruzi* toxin that will allow the exit to the vacuole (Ley et al., 1990). Fusion of the TcPV with early endosomes could explain the mechanism of this acidification through the arrival of subunits of the V-ATPase, which pumps protons from cytosol to the lumen of endocytic compartments. Further interaction with late endosomes through Rab7 and Rab39a would favor the final assembly of the pump (Lafourcade et al., 2008) and the fusion with lysosomes. Vacuole maturation is also characterized by the change in the *T. cruzi* form from the slender trypomastigote to the round-shaped intermediate form indicative that parasite differentiation from trypomastigotes to amastigotes, a process also induced by a reduced pH (Frevert et al., 1995; Romano et al., 2009), is starting inside the vacuole and requires the arrival of late endosomes and lysosomes.

Interestingly, Rabs from the secretory and recycling pathways are absent in the TcPV indicating that these routes do not interact with the *T. cruzi* vacuole at least in the times studied by us. Other authors also showed the absence of Golgi-proteins on nascent parasitophorous vacuoles (Fernandes et al., 2015). In contrast to other pathogens like *C. trachomatis* or *L. pneumophila* that interact with Golgi-derived vesicles to get nutrients for replication (Rejman Lipinski et al., 2009; Schoebel et al., 2009), our data showed that *T. cruzi* do not require components derived from these routes, probably because in the vacuolar stage, *T. cruzi* undergoes the initial steps of differentiation to amastigotes and do not require high quantity of nutrients. In fact, metacyclogenesis, the differentiation of epimastigotes to metacyclic trypomastigotes, performed in the insect stage of the *T. cruzi* cycle, is carried out under nutrient deprivation which activates parasite autophagy, a process required for this differentiation (Vanrell et al., 2017).

The participation of endocytic Rabs on the *T. cruzi* infection was confirmed by the reduction of the infection observed in cells that express the dominant-negative mutants of these Rabs. Although these proteins usually displayed overlapped functions (Zhen and Stenmark, 2015), the impairment of the activity of one of them separately was sufficient to affect the infection. This effect is likely produced by the reduction of functional Rabs available to allow the fusion of endosomes to the nascent vacuoles, which eventually could fuse with the plasma membrane and release the parasites out of cells, in a similar way to the minor infection observed when the lysosomal fusion is impaired (Andrade and Andrews, 2004). Other authors also showed that inhibition of endocytosis processes, when cells are treated with the dynamin inhibitor, dynasore (Barrias et al., 2010) or in the case of LAMP-2 lacking cells where caveolin-dependent endocytosis is impaired (Couto et al., 2017), leads to a reduction on the infection.

In conclusion, this work demonstrated that endocytic Rabs are sequentially acquired by the TcPV and regulate the transit of the vacuole up to the fusion with lysosomes. The impairment of these Rabs affected the fraction of vacuoles that interacts with endosomes and decreased the infection. These data added new molecular regulators on the complex process of *T. cruzi* invasion and contributed to understand the big versatility displayed by *T. cruzi* to parasitize the different types of host cells.

## Data Availability Statement

All datasets generated for this study are included in the article/Supplementary Material.

## Author Contributions

BS, JC, and JG contributed conception and design of experiments, made figures, and performed the statistical analysis. BS and PR contributed conception and design of the study. PR wrote the first draft of the manuscript. BS wrote sections of the manuscript. All authors contributed to manuscript revision, read and approved the submitted version.

## Conflict of Interest

The authors declare that the research was conducted in the absence of any commercial or financial relationships that could be construed as a potential conflict of interest.

## Supplementary Material

Supplementary Figure 1 Recycling and secretory Rabs are not recruited to TcPV. **(A)** CHO cells overexpressing GFP-Rabs (green) were infected 1 h (1hpi) with TCT (MOI 20). After fixation, parasites were detected by indirect immunofluorescence using a specific antibody anti-*T. cruzi* followed by a secondary antibody labeled with Cy3 (red). The DNA of nuclei and kinetoplasts were detected with Hoechst (blue). **(B)** CHO cells overexpressing GFP-Rab6 and GFP-Rab11 were infected for 15 min (15 mpi), 1 or 6 h (hpi) with TCT (MOI 20) and processed as indicated above. The Images are representative for each condition and the magnifications are delimited in the original photo. Scale bar: 10 μm. **(C)** Kinetic graphs represent the recruitment of GFP-Rab6 and GFP-Rab11 to TcPV.

## References

[B1] Andrade L. O. Andrews N. W. (2004). Lysosomal fusion is essential for the retention of *Trypanosoma cruzi* inside host cells. J. Exp. Med. 200, 1135–1143. 10.1084/jem.20041408 15520245 PMC2211867

[B2] Andrade L. O. Andrews N. W. (2005). The *Trypanosoma cruzi*–host-cell interplay: location, invasion, retention. Nat. Rev. Microbiol. 3, 819–823. 10.1038/nrmicro1249 16175174

[B3] Barrias E. S. Reignault L. C. De Souza W. Carvalho T. M. U. (2010). Dynasore, a dynamin inhibitor, inhibits *Trypanosoma cruzi* entry into peritoneal macrophages. PLoS ONE 5:e7764. 10.1371/journal.pone.0007764 20098746 PMC2808331

[B4] Batista D. G. J. Silva C. F. Mota R. A. Costa L. C. Meirelles M. N. L. Meuser-Batista M.. (2006). *Trypanosoma cruzi* modulates the expression of rabs and alters the endocytosis in mouse cardiomyocytes *in vitro*. J. Histochem. Cytochem. 54, 605–614. 10.1369/jhc.5A6654.2005 16009966

[B5] Bucci C. Thomsen P. Nicoziani P. McCarthy J. Van Deurs B. (2000). Rab7: a key to lysosome biogenesis. Mol. Biol. Cell 11, 467–480. 10.1091/mbc.11.2.467 10679007 PMC14786

[B6] Chavrier P. Parton R. G. Hauri H. P. Simons K. Zerial M. (1990). Localization of low molecular weight GTP binding proteins to exocytic and endocytic compartments. Cell 62, 317–329. 10.1016/0092-8674(90)90369-P 2115402

[B7] Chen T. Han Y. Yang M. Zhang W. Li N. Wan T.. (2003). Rab39, a novel Golgi-associated Rab GTPase from human dendritic cells involved in cellular endocytosis. Biochem. Biophys. Res. Commun. 303, 1114–1120. 10.1016/S0006-291X(03)00482-0 12684051

[B8] Christoforidis S. McBride H. M. Burgoyne R. D. Zerial M. (1999). The rab5 effector EEA1 is a core component of endosome docking. Nature 397, 621–625. 10.1038/17618 10050856

[B9] Couto N. F. Pedersane D. Rezende L. Dias P. P. Corbani T. L. Bentini L. C.. (2017). LAMP-2 absence interferes with plasma membrane repair and decreases *T. cruzi* host cell invasion. PLoS Negl. Trop. Dis. 11:e0005657. 10.1371/journal.pntd.0005657 28586379 PMC5473579

[B10] Cueto J. A. Vanrell M. C. Salassa B. N. Nola S. Galli T. Colombo M. I.. (2017). Soluble N-ethylmaleimide-sensitive factor attachment protein receptors required during *Trypanosoma cruzi* parasitophorous vacuole development. Cell. Microbiol. 19, 2–6. 10.1111/cmi.12713 27992096

[B11] Egami Y. Araki N. (2009). Dynamic changes in the spatiotemporal localization of Rab21 in live RAW264 cells during macropinocytosis. PLoS ONE 4:e6689. 10.1371/journal.pone.0006689 19693279 PMC2726762

[B12] Fernandes M. C. Corrotte M. Miguel D. C. Tam C. Andrews N. W. (2015). The exocyst is required for trypanosome invasion and the repair of mechanical plasma membrane wounds. J. Cell Sci. 128, 27–32. 10.1242/jcs.150573 25380822 PMC4282046

[B13] Fernandes M. C. Cortez M. Flannery A. R. Tam C. Mortara R. A. Andrews N. W. (2011). *Trypanosoma cruzi* subverts the sphingomyelinase-mediated plasma membrane repair pathway for cell invasion. J. Exp. Med. 208, 909–921. 10.1084/jem.20102518 21536739 PMC3092353

[B14] Frevert U. Vandekerckhove F. Nussenzweig V. (1995). The induction of *Trypanosoma cruzi* trypomastigote to amastigote transformation by low pH. Parasitology 110, 547–554. 10.1017/S0031182000065264 7541124

[B15] Gambarte Tudela J. Buonfigli J. Luján A. Alonso Bivou M. Cebrián I. Capmany A.. (2019). Rab39a and Rab39b display different intracellular distribution and function in sphingolipids and phospholipids transport. Int. J. Mol. Sci. 20:1688. 10.3390/ijms20071688 30987349 PMC6480249

[B16] Gorvel J. P. Chavrier P. Zerial M. Gruenberg J. (1991). rab5 controls early endosome fusion *in vitro*. Cell 64, 915–925. 10.1016/0092-8674(91)90316-Q 1900457

[B17] Grant B. D. Donaldson J. G. (2009). Pathways and mechanisms of endocytic recycling. Nat. Rev. Mol. Cell Biol. 10, 597–608. 10.1038/nrm2755 19696797 PMC3038567

[B18] Kumar Y. Valdivia R. H. (2009). Leading a sheltered life: intracellular pathogens and maintenance of vacuolar compartments. Cell Host Microbe 5, 593–601. 10.1016/j.chom.2009.05.014 19527886 PMC2716004

[B19] Lafourcade C. Sobo K. Kieffer-Jaquinod S. Garin J. van der Goot F. G. (2008). Regulation of the V-ATPase along the endocytic pathway occurs through reversible subunit association and membrane localization. PLoS ONE 3:e2758. 10.1371/journal.pone.0002758 18648502 PMC2447177

[B20] Ley V. Robbins E. S. Nussenzweig V. Andrews N. W. (1990). The exit of *Trypanosoma cruzi* from the phagosome is inhibited by raising the pH of acidic compartments. J. Exp. Med. 171, 401–413. 10.1084/jem.171.2.401 2406362 PMC2187728

[B21] Li G. (2012). Early endocytosis: Rab5, rab21, and rab22, in Rab GTPases and Membrane Trafficking, eds Li G. Segev N. (Sharjah: Bentham Science Publishers Ltd), 93–107.

[B22] Magadán J. G. Barbieri M. A. Mesa R. Stahl P. D. Mayorga L. S. (2006). Rab22a regulates the sorting of transferrin to recycling endosomes. Mol. Cell Biol. 26, 2595–2614. 10.1128/MCB.26.7.2595-2614.2006 16537905 PMC1430328

[B23] Maganto-Garcia E. Punzon C. Terhorst C. Fresno M. (2008). Rab5 activation by toll-like receptor 2 is required for *trypanosoma cruzi* internalization and replication in macrophages. Traffic 9, 1299–1315. 10.1111/j.1600-0854.2008.00760.x 18445119 PMC4482123

[B24] Martinez-Arca S. Alberts P. Galli T. (2000a). Clostridial neurotoxin-insensitive vesicular SNAREs in exocytosis and endocytosis. Biol. Cell 92, 449–453. 10.1016/S0248-4900(00)01096-0 11132707

[B25] Martinez-Arca S. Alberts P. Zahraoui A. Louvard D. Galli T. (2000b). Role of tetanus neurotoxin insensitive vesicle-associated membrane protein (TI-VAMP) in vesicular transport mediating neurite outgrowth. J. Cell Biol. 149, 889–899. 10.1083/jcb.149.4.889 10811829 PMC2174569

[B26] Rejman Lipinski A. Heymann J. Meissner C. Karlas A. Brinkmann V. Meyer T. F.. (2009). Rab6 and Rab11 regulate *Chlamydia trachomatis* development and golgin-84-dependent Golgi fragmentation. PLoS Pathog. 5:e1000615. 10.1371/journal.ppat.1000615 19816566 PMC2752117

[B27] Rieder S. E. Emr S. D. (1997). A novel RING finger protein complex essential for a late step in protein transport to the yeast vacuole. Mol. Biol. Cell 8, 2307–2327. 10.1091/mbc.8.11.2307 9362071 PMC25710

[B28] Rink J. Ghigo E. Kalaidzidis Y. Zerial M. (2005). Rab conversion as a mechanism of progression from early to late endosomes. Cell 122, 735–749. 10.1016/j.cell.2005.06.043 16143105

[B29] Rivera-Molina F. E. Novick P. J. (2009). A Rab GAP cascade defines the boundary between two Rab GTPases on the secretory pathway. *Proc. Natl. Acad. Sci. U.S*. A. 106, 14408–14413. 10.1073/pnas.0906536106 19666511 PMC2732877

[B30] Rodríguez A. Samoff E. Rioult M. G. Chung A. Andrews N. W. (1996). Host cell invasion by trypanosomes requires lysosomes and microtubule/kinesin-mediated transport. J. Cell Biol. 134, 349–362. 10.1083/jcb.134.2.349 8707821 PMC2120885

[B31] Romano P. S. Arboit M. A. Vázquez C. L. Colombo M. I. (2009). The autophagic pathway is a key component in the lysosomal dependent entry of *Trypanosoma cruzi* into the host cell. Autophagy 5, 6–18. 10.4161/auto.5.1.7160 19115481

[B32] Savina A. Vidal M. Colombo M. I. (2002). The exosome pathway in K562 cells is regulated by Rab11. J. Cell Sci. 115 (Pt. 12), 2505–2515. 12045221 10.1242/jcs.115.12.2505

[B33] Schlüter O. M. Schmitz F. Jahn R. Rosenmund C. Südhof T. C. (2004). A complete genetic analysis of neuronal Rab3 function. J. Neurosci. 24, 6629–6637. 10.1523/JNEUROSCI.1610-04.2004 15269275 PMC6729882

[B34] Schoebel S. Oesterlin L. K. Blankenfeldt W. Goody R. S. Itzen A. (2009). RabGDI displacement by DrrA from legionella is a consequence of its guanine nucleotide exchange activity. Mol. Cell 36, 1060–1072. 10.1016/j.molcel.2009.11.014 20064470

[B35] Seals D. F. Eitzen G. Margolis N. Wickner W. T. Price A. (2000). A Ypt/Rab effector complex containing the Sec1 homolog Vps33p is required for homotypic vacuole fusion. Proc. Natl. Acad. Sci. U.S.A. 97, 9402–9407. 10.1073/pnas.97.17.9402 10944212 PMC16876

[B36] Simpson J. C. Griffiths G. Wessling-Resnick M. Fransen J. A. M. Bennett H. Jones A. T. (2004). A role for the small GTPase Rab21 in the early endocytic pathway. J. Cell Sci. 117, 6297–6311. 10.1242/jcs.01560 15561770

[B37] Tardieux I. Webster P. Ravesloot J. Boron W. Lunn J. A. Heuser J. E.. (1992). Lysosome recruitment and fusion are early events required for trypanosome invasion of mammalian cells. Cell 71, 1117–1130. 10.1016/S0092-8674(05)80061-3 1473148

[B38] Tisdale E. J. Bourne J. R. Khosravi-Far R. Der C. J. Balch W. E. (1992). GTP-binding mutants of Rab1 and Rab2 are potent inhibitors of vesicular transport from the endoplasmic reticulum to the golgi complex. J. Cell Biol. 119, 749–761. 10.1083/jcb.119.4.749 1429835 PMC2289685

[B39] Vanrell M. C. Losinno A. D. Cueto J. A. Balcazar D. Fraccaroli L. V. Carrillo C.. (2017). The regulation of autophagy differentially affects *Trypanosoma cruzi* metacyclogenesis. PLoS Negl. Trop. Dis. 11:e0006049. 10.1371/journal.pntd.0006049 29091711 PMC5683653

[B40] Wandinger-Ness A. Zerial M. (2014). Rab proteins and the compartmentalization of the endosomal system. Cold Spring Harb. Perspect. Biol. 6:a022616. 10.1101/cshperspect.a022616 25341920 PMC4413231

[B41] Wilkowsky S. E. Barbieri M. A. Stahl P. D. Isola E. L. D. (2002). Regulation of *Trypanosoma cruzi* invasion of nonphagocytic cells by the endocytically active GTPases dynamin, Rab5, and Rab7. Biochem. Biophys. Res. Commun. 291, 516–521. 10.1006/bbrc.2002.6474 11855818

[B42] Woolsey A. M. Burleigh B. A. (2004). Host cell actin polymerization is required for cellular retention of *Trypanosoma cruzi* and early association with endosomal/lysosomal compartments. Cell Microbiol. 6, 829–838. 10.1111/j.1462-5822.2004.00405.x 15272864

[B43] Woolsey A. M. Sunwoo L. Petersen C. A. Brachmann S. M. Cantley L. C. Burleigh B. A. (2003). Novel PI 3-kinase-dependent mechanisms of trypanosome invasion and vacuole maturation. J. Cell Sci. 116, 3611–3622. 10.1242/jcs.00666 12876217

[B44] Xu H. Ren D. (2015). Lysosomal physiology. Annu. Rev. Physiol. 77, 57–80. 10.1146/annurev-physiol-021014-071649 25668017 PMC4524569

[B45] Zerial M. Stenmark H. (1993). Rab GTPases in vesicular transport. Curr. Opin. Cell Biol. 5, 613–620. 10.1016/0955-0674(93)90130-I 8257602

[B46] Zhen Y. Stenmark H. (2015). Cellular functions of Rab GTPases at a glance. J. Cell Sci. 128, 3171–3176. 10.1242/jcs.166074 26272922

[B47] Zhu H. Liang Z. Li G. (2009). Rabex-5 is a rab22 effector and mediates a rab22-rab5 signaling cascade in endocytosis. Mol. Biol. Cell 20, 4720–4729. 10.1091/mbc.e09-06-0453 19759177 PMC2777102

