# Supplementary figures and images for: Endocytic Rabs Are Recruited to the Trypanosoma cruzi Parasitophorous Vacuole and Contribute to the Process of Infection in Non-professional Phagocytic Cells
Source: Front Cell Infect Microbiol. 2020 Oct 29;10:536985. doi: 10.3389/fcimb.2020.536985 (PMC7658340; doi:10.3389/fcimb.2020.536985)

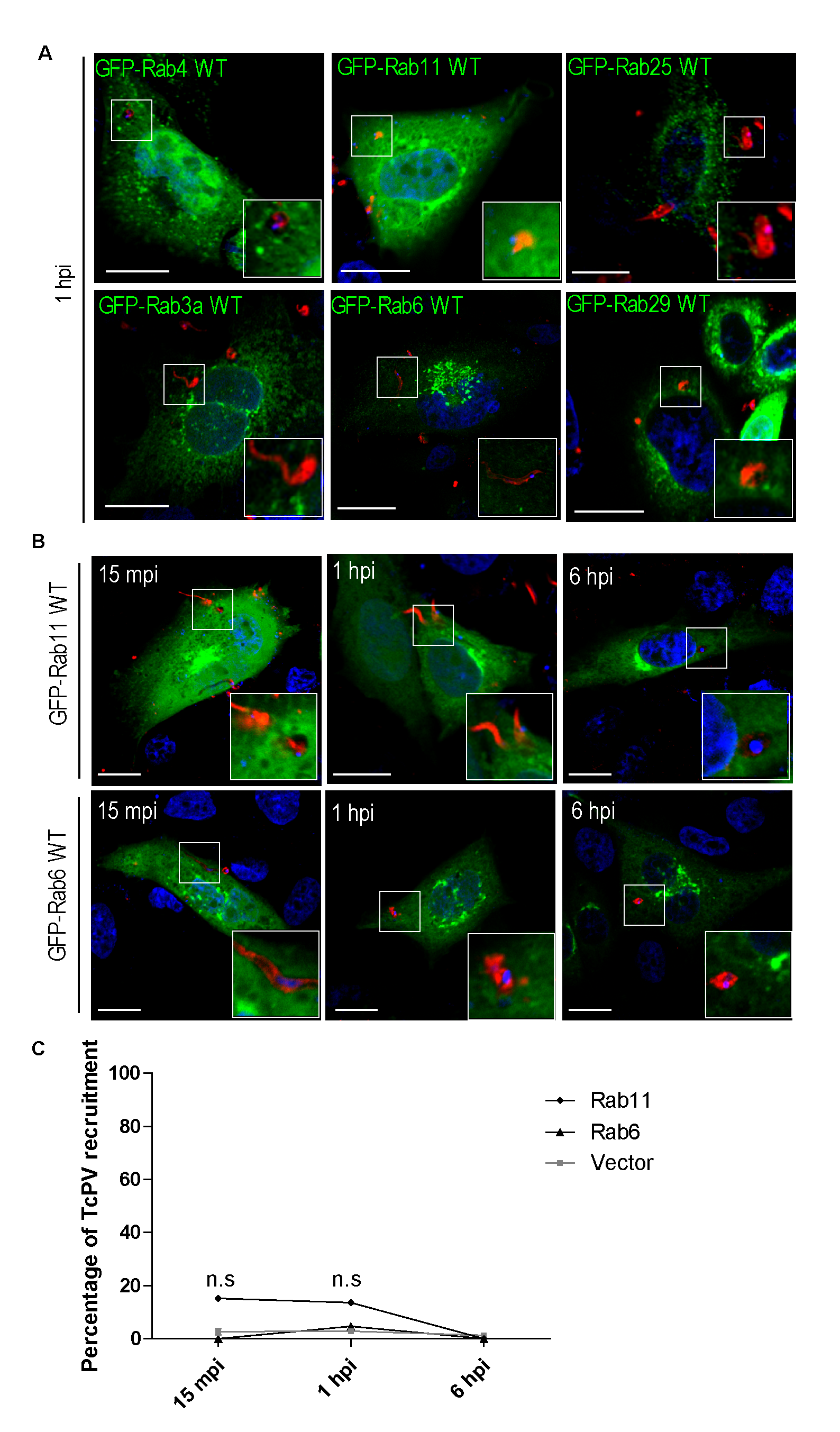

Supplement: Supplementary Figure 1 — Recycling and secretory Rabs are not recruited to TcPV. (A) CHO cells overexpressing GFP-Rabs (green) were infected 1 h (1hpi) with TCT (MOI 20). After fixation, parasites were detected by indirect immunofluorescence using a specific antibody anti-T. cruzi followed by a secondary antibody labeled with Cy3 (red). The DNA of nuclei and kinetoplasts were detected with Hoechst (blue). (B) CHO cells overexpressing GFP-Rab6 and GFP-Rab11 were infected for 15 min (15 mpi), 1 or 6 h (hpi) with TCT (MOI 20) and processed as indicated above. The Images are representative for each condition and the magnifications are delimited in the original photo. Scale bar: 10 μm. (C) Kinetic graphs represent the recruitment of GFP-Rab6 and GFP-Rab11 to TcPV. [file Image_1.TIF]
